# Long-Term Durability of Active Surveillance of Small, Low-Risk Papillary Thyroid Cancer

**DOI:** 10.1001/jamasurg.2025.2957

**Published:** 2025-08-20

**Authors:** Anna M. Sawka, Sangeet Ghai, Lorne Rotstein, Jonathan C. Irish, Jesse D. Pasternak, Eric Monteiro, Janet Chung, Jie Su, Wei Xu, Alex O. Esemezie, Jennifer M. Jones, Amiram Gafni, Nancy N. Baxter, David P. Goldstein

**Affiliations:** 1 Division of Endocrinology, University Health Network and University of Toronto, Toronto, Ontario, Canada; 2 Joint Department of Medical Imaging, University Health Network–Mt Sinai Hospital–Women’s College Hospital, University of Toronto, Toronto, Ontario, Canada; 3 Department of Surgery, University Health Network and University of Toronto, Toronto, Ontario, Canada; 4 Department of Otolaryngology–Head and Neck Surgery/Surgical Oncology, Princess Margaret Cancer Centre, University Health Network, Toronto, Ontario, Canada; 5 Department of Otolaryngology and Head and Neck Surgery, Mount Sinai Hospital and University of Toronto, Toronto, Ontario, Canada; 6 Department of Otolaryngology and Head and Neck Surgery, Trillium Health Partners and University of Toronto, Mississauga, Ontario, Canada; 7 Department of Biostatistics, Princess Margaret Cancer Centre, University Health Network, Toronto, Ontario, Canada; 8 Department of Biostatistics, Dalla Lana School of Public Health, University of Toronto, Toronto, Ontario, Canada; 9 Department of Supportive Care, University Health Network and University of Toronto, Toronto, Ontario, Canada; 10 Centre for Health Economics and Policy Analysis, Department of Health Research Methods, Evidence, and Impact, McMaster University, Hamilton, Ontario, Canada; 11 Faculty of Medicine and Health University of Sydney, Sydney, New South Wales, Australia

## Abstract

**Importance:**

In managing early-stage cancers, active surveillance (AS) may be preferentially favored by older individuals. In counseling patients, it is important to understand the durability of AS in the context of age.

**Objective:**

To evaluate the durability of AS in patients with small, low-risk papillary thyroid cancer (PTC) according to age at the time of choosing AS.

**Design, Setting, and Participants:**

This single-center, prospective, long-term follow-up cohort study was conducted at a tertiary care hospital in Toronto, Ontario, Canada. Adult patients with small, localized, low-risk PTC less than 2 cm in maximal diameter were enrolled between May 2016 and February 2021. The clinical outcome data were analyzed up to the time point of May 25, 2025, and final data analysis was performed in June 2025.

**Exposure:**

All patients were offered the choice of AS or thyroid surgery.

**Main Outcomes and Measures:**

The primary outcome was the overall rate of AS crossover to definitive treatment (treatment completed or recommended by an investigator) and the indications. Cumulative crossover incidence function curves were examined according to age, with death from other causes as the competing risk.

**Results:**

A total of 200 patients (155 patients under AS and 45 who had immediate surgery) were followed up for a median (IQR) duration of 71 (59-84) months. Overall mean (SD) age was 52.0 (14.9) years, and 153 patients (76.5%) were female. There were no observed thyroid cancer–related deaths or any distant metastatic disease. The overall crossover rate from AS was 23.9% (37/155; 32 completed treatment, 3 declined surgery for disease progression, and 2 awaiting treatment). Crossover reasons included disease progression (56.8% [21/37]), patient preference (40.5% [15/37]), and ultrasound imaging limitations precluding accurate tumor measurement under active surveillance (tumor border not clearly distinguishable from heterogeneous echotexture of the thyroid parenchyma in a patient with Hashimoto thyroiditis; 2.6% [1/37]). The 5-year age-stratified cumulative overall crossover incidence rates were 41.5% (95% CI, 25.6%-56.8%) in patients younger than 45 years, 20.9% (95% CI, 12.3%-31.1%) in those aged 45 to 64 years, and 5.1% (95% CI, 0.9%-15.2%) in those aged 65 years and older (*P* < .001).

**Conclusion and Relevance:**

This single-center Canadian cohort study found that AS is a durable long-term management strategy for small, low -risk PTC, particularly in older individuals. Older individuals may be less likely to cross over to surgery after choosing AS.

## Key Points

**Question:**

Does the durability of active surveillance (AS) of low-risk papillary thyroid cancer (PTC) vary by age?

**Findings:**

In this cohort study of patients with small, low-risk PTC, 118 of 155 patients (76%) who chose AS continued AS in the absence of disease progression or other indications for surgery at long-term follow-up (median [IQR] follow-up duration, 71 [59-84] months). The 5-year rates of continuing AS were 94.9% for patients aged 65 years or older, 79.1% for those aged 45 to 64 years, and 58.5% for those younger than 45 years.

**Meaning:**

The long-term durability of AS for small, low-risk PTC may be greater in patients of older age.

## Teaser

This single-center cohort study evaluates the durability of active surveillance in patients with small, low-risk papillary thyroid cancer according to age at the time of choosing active surveillance.

## Introduction

Active surveillance (AS) of cancer is an attractive disease management option for slow-growing neoplasms, where the risk of dying from the tumor is low, and where the patient may be closely monitored, with a plan for timely curative treatment in the event of disease progression. AS is especially suitable for patients with significant medical comorbidities for whom the treatment itself may be associated with significant risk. AS is thus intended to reduce the burden of treatment-related morbidity and potentially preserve general health and quality of life in patients at low oncologic risk for whom the psychological consequence of living with their tumor is acceptable. There is emerging evidence that AS of neoplasms may be preferentially adopted by older individuals, as reflected in studies of prostate cancer,^1^ early-stage lung cancer,^2^ and papillary thyroid cancer (PTC).^3^ However, the durability of AS is not well understood, particularly as it relates to patient age. Long-term AS durability would depend on how often the neoplasm under AS progresses and how often patients change their mind about AS and decide to have active treatment in absence of disease progression, as well as competing risks of death.

The current general standard of care for primary management of PTC is thyroidectomy.^4^ However, given the generally excellent prognosis of small, low-risk PTC, there has been growing expert support for considering more conservative treatment approaches to minimize the potential treatment-related morbidities (eg, risks of hypoparathyroidism, recurrent laryngeal nerve injury, bleeding, anesthetic risks, and lifelong thyroid hormone treatment).^4^ AS, therefore, is an attractive alternative primary disease management strategy for small, low-risk PTC. Investigators from Kuma Hospital in Japan have reported a favorable 30-year experience using this approach in patients with papillary microcarcinoma(s) (measuring ≤1 cm in maximal diameter).^5^ There is substantial confirmatory observational research on the safety of AS in papillary microcarcinoma, but knowledge gaps remain in the outcomes of AS for patients with tumors larger than 1 cm in maximal diameter, as well as in older patients.^6^

We initiated a prospective observational cohort study in Toronto, Canada, in 2016 offering adult patients (aged ≥18 years) with low-risk PTC less than 2 cm in maximal diameter the option of AS or surgery (NCT03271892).^7^ We observed that older age was significantly independently associated with choosing AS over initial surgery, such that compared to individuals younger than 40 years, the odds ratio of choosing AS over surgery was 2.78 in patients aged 40 to 64 years (*P* = .01) and 8.43 in those aged 65 years and older (*P* = .002), regardless of primary tumor size.^3^ There is published observational evidence suggesting that older age may be associated with a reduced risk of PTC progression under AS, largely based on natural history of tumor growth over time.^8,9,10,11,12^ In this analysis of prospective observational cohort data, we explored the durability of patients’ choice of AS (strictly defined as continuing AS in the absence of a crossover indication) according to patient age at the time of enrollment.

## Methods

The original protocol of this study (NCT03271892)^7^ and study amendments (largely in response to the COVID-19 pandemic) have been previously reported in detail.^13^ The University Health Network Research Ethics Board approved this study (15-8942) and all patients provided written informed consent for study participation.

### Participant Inclusion and Exclusion Criteria

We included patients aged 18 years or older with cytologic evidence of PTC or suspected PTC measuring less than 2 cm in maximal diameter.^7^ Exclusion criteria included presence of known metastatic PTC in lymph nodes or distant sites, evidence of extrathyroidal extension of the PTC, or PTC deemed at high risk of tracheal or recurrent laryngeal invasion (based on location and configuration of the tumor relative to the trachea or nerve).^7^ Patients who had an absolute indication for thyroid or parathyroid surgery that was not related to thyroid cancer were not eligible for the study.^13^ Patients were enrolled between May 2016 and February 2021 at the University Health Network in Toronto, Canada.

### Study Procedures and Data Collection

Patients were offered the choice of AS or surgery; patients who chose surgery or those who crossed over to surgery from AS underwent usual care as per their surgeon, endocrinologist, and other physicians. The AS study protocol included ultrasound examinations in the joint departmental medical imaging department, blood work (thyrotropin, thyroglobulin, and thyroglobulin antibody levels), and clinical assessment every 6 months for 2 years, followed by yearly assessment if stable.^7^ Participants’ medical records were reviewed at baseline and at least yearly.^7^ The clinical outcome data were analyzed up to the time point of May 25, 2025. Patients in this study continue to be followed up as part of a larger multicenter study.^14^

### Study Outcomes

For this analysis, the primary outcome was the overall rate of crossover from AS to surgical treatment or localized thermal ablation treatment (the latter if obtained by patients outside of the study). The overall crossover rate included patients who underwent thyroidectomy or localized thermal ablation treatment (outside the study) or those who received a recommendation for surgical treatment for disease progression or related concern from a study investigator (regardless of having received the treatment in the case of patients who declined it or were awaiting treatment). The type of treatment recommended in the study for disease progression was thyroid surgery, but if a patient under AS chose to cross over and undergo localized thermal ablation therapy outside the study (with or without disease progression), we continued to follow up the patient and report the long-term outcomes. For the purpose of estimation of the overall crossover rate, the use of localized thermal ablation therapy was counted as a form of crossover to active treatment.

We described the long-term outcomes of patients who chose AS to those who underwent immediate surgery, including overall and disease-specific mortality, as well as incidence of distant metastatic disease and disease status at the time of last follow-up. We also described the type of thyroid cancer surgery performed and any radioactive iodine treatment.

### Statistical Analyses

We included all available data from participants until last follow-up as of the data cutoff date. Descriptive data are presented on patient characteristics and patient outcomes according to the initial choice of AS or surgery. We used the χ^2^ or Fisher exact test for group comparisons of categorical data. Continuous data were compared using the Wilcoxon rank sum test. We reported means and standard deviations, as well as medians and interquartile ranges (quartiles 1 and 3) for continuous data and frequency with percentages for categorical data.

The rates of overall crossover (and the subgroups of crossover due to disease progression and crossover due to patient preference) were compared according to participant age category at the time of enrollment (<45, 45-64 years, and ≥65 years) for patients who had chosen AS. The rationale for the age category of less than 45 years is that age less than 40 to 50 years was found to be associated with an increased risk of PTC progression in a prior systematic review conducted by our group^8^ and typically, for females, childbearing (if desired) occurs during that time frame. The age of 65 years was chosen as this is considered the traditional retirement age in Canada and is commonly used as a cut point for health care analysis and reporting. As for the category of 45 to 64 years, this was considered middle age, between the other 2 categories.

Time to crossover was calculated from the date of enrollment to the date of crossover treatment or the date of last follow-up. Crossover treatment was the specific event, and nonthyroid cancer death without crossover was considered the competing event for the analysis. The cumulative incidence function was used to estimate the 5-year cumulative incidence rate of overall crossover among age categories (<45, 45-64, and ≥65 years). We reported 95% confidence intervals for the cumulative incidence rates. The Gray test was used to compare the overall crossover cumulative incidence rates among age categories.

A secondary multivariable Fine-Gray competing risk regression analysis was performed, examining associations between participant age at enrollment (continuous variable), primary tumor size at baseline, primary tumor cytology at baseline, and level of education (postgraduate or professional degree vs university, college, or high school or lower), with the outcome of time to overall crossover (ie, crossover or meeting indications for crossover). The rationale for including these variables included consideration of the clinical importance of age, tumor size, and cytology, as well as our prior observation of an association between lower education and choosing AS.^3^ In a post hoc exploratory analysis, this multivariable model was used to identify the optimal age cutoff associated with crossover. The optimal age cutoff was identified by maximizing log-likelihood in this multivariable model. The results of the time-to-event analyses were expressed as hazard ratios with 95% confidence internals. In secondary analyses, we also compared the main reasons for crossover from AS to treatment (ie, disease progression or patient preference) according to age category.

Missing data were excluded, and the statistical significance threshold for all analyses was defined as *P* < .05 (2-sided). The statistical analyses were performed using R version 4.3.1 (R Foundation) and SAS version 9.4 (SAS Institute).

## Results

### Study Participant Characteristics and General Long-Term Outcomes

**Figure 1.**
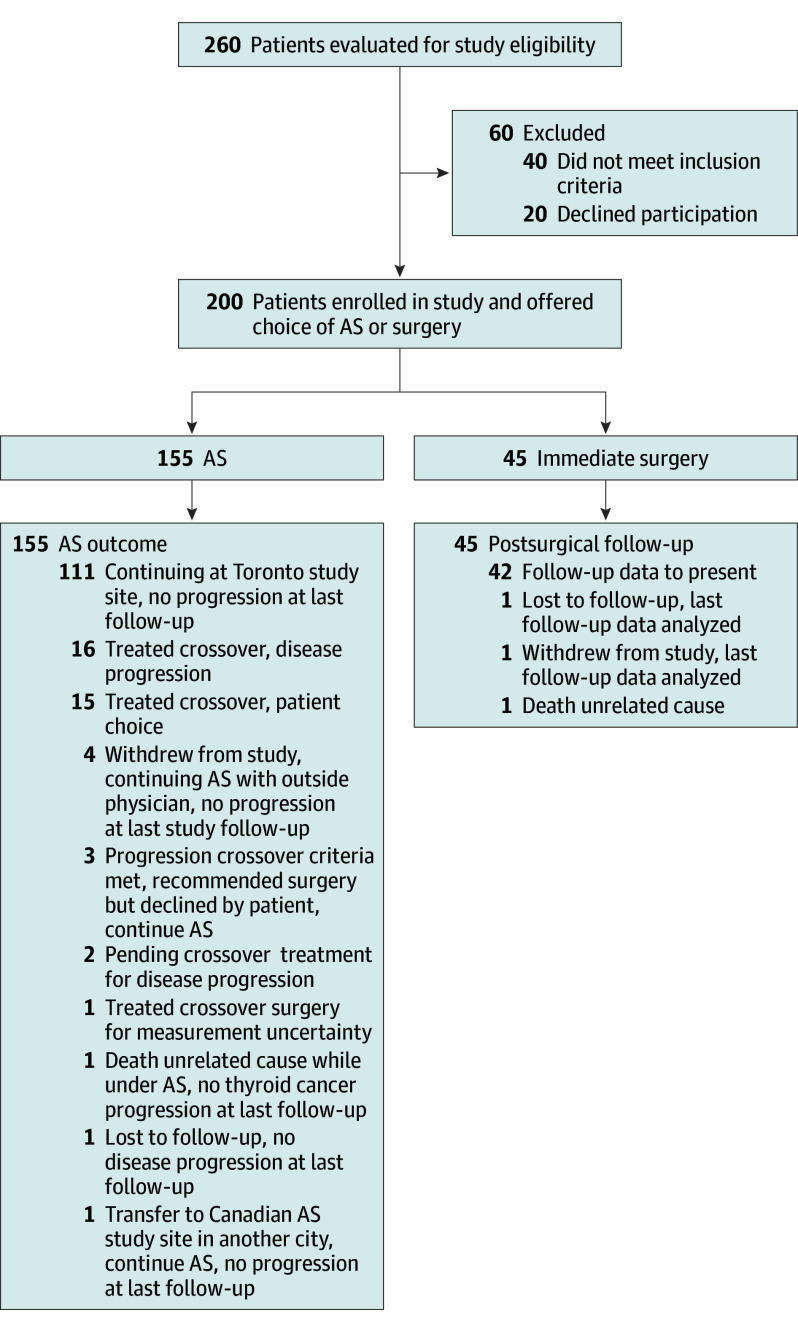
Participant Flow Diagram AS indicates active surveillance

We enrolled 153 female patients (76.5%) and 47 male patients (23.5%), with overall mean (SD) age of 52.0 (14.9) years. Of 200 patients, 155 (78%) chose AS, and the remainder (45/200) chose surgery. The median (IQR) duration of follow-up for the entire study population was 71 (59-84) months. The median (IQR) duration of follow-up under AS was 66 (53-79) months. A detailed participant flow diagram is shown in Figure 1. None of the patients died of thyroid cancer. One patient in the AS group (1/155 [0.6%]) and 1 in the surgical group (1/45 [2.2%]) died of unrelated causes during the study. None of the study participants were diagnosed with any distant metastatic thyroid cancer during follow-up. The details of study participant characteristics according to whether the individual chose AS or surgery are shown in Table 1, and additional details were previously published.^3^ The frequency with which AS was chosen over surgery varied significantly by age at enrollment, such that older patients were more likely to choose to undergo AS than younger patients (Table 1).

**Table 1.** Study Participant Characteristics Grouped According to Initial Disease Management Choice

Variable	No./total No. (%)		*P* value
	Active surveillance (n = 155)	Immediate surgery (n = 45)	
Sex			
Female	120/155 (77)	33/45 (73)	.71
Male	35/155 (23)	12/45 (27)	
Age category at enrollment, y			
<45 (n = 64)	40/155 (26)	24/45 (53)	<.001
45-64 (n = 93)	75/155 (48)	18/45 (40)	
≥65 (n = 43)	40/155 (26)	3/45 (7)	
Primary tumor size at baseline, mean (SD), mm	11.1 (3.8)	12.3 (3.5)	.06
Primary tumor size category at baseline, cm maximal diameter			
≤1	73/155 (47)	15/45 (33)	.14
>1	82/155 (53)^a^	30/45 (67)	
Baseline thyrotropin, median (IQR), mIU/L	1.46 (1.02-2.17)	1.89 (1.15-2.42)	.18
Taking thyroid hormone treatment at time of enrollment	19/155 (12)	9/45 (20)	.28
Duration of follow-up, median (IQR), mo	69 (58-83)	77 (66-90)	.05

^a^ Primary tumor size measurement performed at baseline, and baseline measurement reassessed in follow-up for patients under active surveillance if concern about possible tumor growth (to enable quantification of the growth).

### Overall Crossover Rate From AS to Surgery

The overall crossover rate from AS to treatment was 23.9% (37/155, including 32 patients who completed treatment [31 undergoing surgery and 1 who sought out radiofrequency ablation (RFA) outside of the study within a month of enrollment due to patient choice], 3 who declined surgery for disease progression, and 2 awaiting treatment). The reasons for crossover included disease progression (56.8% [21/37]), patient preference (40.5% [15/37], including the 1 patient that underwent RFA), and ultrasound imaging limitations precluding accurate tumor measurement under active surveillance (ie, tumor border not clearly distinguishable from heterogeneous echotexture of the thyroid parenchyma in a patient with Hashimoto thyroiditis) (2.6% [1/37]). The median (IQR) duration of active surveillance according to crossover indication was 50 (37-56) months for disease progression (in 21 patients), 12 (7-37) months for patient preference (in 15 patients), and 25 months for investigator-directed crossover due to measurement uncertainty (1 patient). Of the 21 patients meeting crossover indications for tumor progression, 1 patient (4.8%) had incident nodal metastatic disease, 5 (23.8%) had suspected extrathyroidal extension, and 15 (71.4%) had tumor enlargement of either the primary tumor (n = 13) or another PTC that was not the original index primary tumor (n = 2).

**Figure 2.**
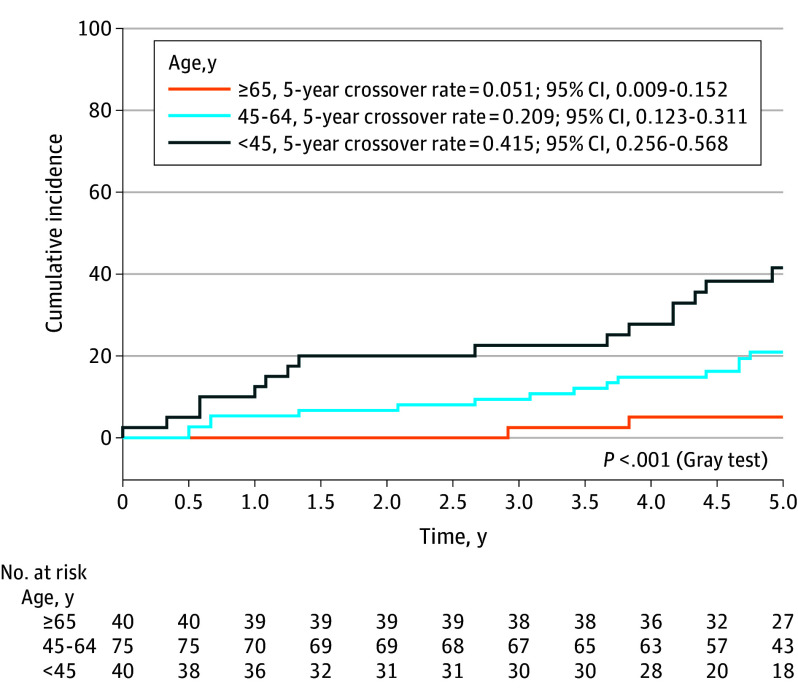
Cumulative Incidence Function Curve for Overall Crossover From Active Surveillance to Active Treatment^a^ ^a^Death from causes other than thyroid cancer is the competing risk (n = 1).

The median (IQR) time from enrollment to definitive treatment of PTC in patients who crossed over from AS was 34 (11-47) months for the 32 patients who received treatment as of the time of the data cutoff for analysis. The 5-year cumulative overall crossover incidence rates according to age at study enrollment were 41.5% (95% CI, 25.6%-56.8%) for patients younger than 45 years, 20.9% (95% CI, 12.3%-31.1%) for those aged 45 to 64 years, and 5.1% (95% CI, 0.9%-15.2%) for those aged 65 years and older (*P* < .001; Figure 2). The AS crossover rates for disease progression did not vary significantly by age categories: 20% (8/40) for patients younger than 45 years, 13% (10/75) for those aged 45 to 64 years, and 8% (3/40) for those 65 years and older (*P* = .28). However, patient preference–directed crossover rates were significantly age dependent: 23% (9/40) for patients younger than 45 years, 8% (6/75) for those aged 45 to 64 years, and 0% for those 65 years and older (*P* = .002). The characteristics of patients who chose AS and crossed over to active treatment (or met criteria for crossover) compared to those who did not cross over are compared in Table 2.^15^ Age was independently associated with time to crossover in a multivariable model that accounted for tumor size, baseline cytology, and education (Table 3).^15^ In an exploratory analysis, the optimal age cutoff identified by maximizing log-likelihood in this multivariable model was 55 years.

**Table 2.** Comparison of the Characteristics of Patients Under Active Surveillance Who Crossed Over (or Met Study Crossover Criteria) Compared to Those Who Did Not Cross Over^a^

Variable	No./total No. (%)		*P* value
	Active surveillance: overall crossover to active treatment (n = 37)^a^	Active surveillance: no crossover (n = 118)	
Sex			
Female	29/37 (78)	91/118 (77)	>.99
Male	8/37 (22)	27 (23)	
Age category at enrollment, y			
<45 (n = 40)	17/37 (46)	23/118 (19)	.001
45-64 (n = 75)	17/37 (46)	58/118 (49)	
≥65 (n = 40)	3/37 (8)	37/118 (31)	
Highest level of education achieved			
≤High school degree	8/37 (22)	39/118 (33)	.04
University or college degree	18/37 (49)	64/118 (54)	
Postgraduate of professional degree	11/37 (30)	15/118 (13)	
Primary tumor size at baseline, mean (SD), mm	12.0 (3.6)	10.8 (3.9)	.10
Baseline primary tumor size category, cm maximal diameter			
≤1	15/37 (41)	58/118 (49)	.47
>1	22/37 (59)	60/118 (51)	
Baseline primary tumor cytology			
Positive for malignancy (Bethesda VI)^b^	23/37 (62)	90/118 (76)	.14
Suspicious for malignancy (Bethesda V)^b^	14/37 (38)	28/118 (24)	
Thyrotropin level at baseline, median (IQR), mIU/L	1.60 (1.07-2.18)	1.38 (1.02-2.16)	.65
Duration under active surveillance, median (IQR), mo	41 (13-53)	69 (58-83)	<.001
Thyrotropin level at last follow-up, median (IQR), mIU/L^c^	1.51 (0.76-2.26)	1.56 (0.96-2.07)	.25
Duration of study follow-up, median (IQR), mo	69 (58-82)	69 (58-83)	.97

^a^ Overall crossover is defined as patients who initially chose active surveillance and ultimately underwent surgery/definitive treatment or received a recommendation for surgery from a study investigator (regardless of having received the treatment, in the case of patients who declined it or were awaiting treatment).

^b^ Categories as per the 2023 Bethesda System for Reporting Thyroid Cytopathology.^15^ Positive for malignancy and suspicious for malignancy refer to papillary thyroid carcinoma.

^c^ 32 Patients in the crossover group, 117 in the no-crossover group.

**Table 3.** Fine-Gray Competing Risk Multivariable Regression Analysis Examining Association With Time to Overall Crossover (Treatment Completed or Recommended by an Investigator) in Patients Who Initially Chose Active Surveillance^a^

Variable	Hazard ratio (95% CI)	*P* value
Age at baseline	0.96 (0.94-0.98)	<.001
Primary tumor size at baseline	1.03 (0.97-1.11)	.32
Primary tumor cytology at baseline		
Positive for malignancy (Bethesda VI)^b^	1 [Reference]	.06
Suspicious for malignancy (Bethesda V)^b^	1.98 (0.98-4.00)	
Education		
≤College/university	1 [Reference]	.03
Postgraduate/professional degree	2.30 (1.11-4.77)	

^a^ This analysis includes all 155 patients who chose AS. There were 37 crossover events included.

^b^ Categories as per the 2023 Bethesda System for Reporting Thyroid Cytopathology.^15^ Positive for malignancy and suspicious for malignancy refer to papillary thyroid carcinoma.

### Treatment Outcomes

Most patients in both the AS crossover group and the initial surgical group underwent hemithyroidectomy without any cervical nodal dissection, and there were no significant differences in either the extent of surgery or use of radioactive iodine treatment in these groups (eTable in Supplement 1).^16^ Furthermore, treatment complications were very infrequent and not significantly different between patients who crossed over from AS to definitive treatment compared to those who had initial surgery (eTable in Supplement 1).^16^ Regardless of whether the initial choice was AS or surgery, most treated patients were disease-free at last follow-up (eTable in Supplement 1).^16^

## Discussion

In this prospective cohort study offering patients with small, low-risk PTC the choice of AS or surgery, approximately 3 of 4 patients who chose AS continued it in absence of disease progression or other indications for surgery over a median follow-up period of 71 months. We found that older patients with low-risk PTC were more likely to choose AS over surgery compared to younger patients,^3^ and, in this study, the long-term durability of AS was greater in older patients. The likelihood of continuing AS 5 years after its initiation (in absence of disease progression or changing one’s mind) was about 95% in those aged 65 years and older, 79% for those aged 45 to 64 years, and 59% in individuals younger than 45 years.

The long-term oncologic outcomes in our study were highly favorable. This is particularly important given the cytologic confirmation of the diagnosis in all patients at baseline, the relatively long duration of prospectively collected follow-up data, and the fact that more than half the study population was from an understudied group of patients with PTC larger than 1 cm in maximal diameter (ie, larger than microcarcinoma) at the time of enrollment. None of the patients in the study died of thyroid cancer or developed distant metastatic thyroid cancer. Furthermore, the long-term oncologic outcomes and the nature of treatments of patients who crossed over from AS to surgery in this study did not significantly differ from patients who had chosen immediate surgery (albeit the small sample sizes). Our data compare favorably relative to other cohort studies examining AS outcomes in patients with small, low-risk PTC where investigators have included some patients with tumors larger than 1 cm in maximal diameter,^10,17,18,19,20,21^ particularly considering differences in study design and follow-up duration. Thus, there is growing evidence on the safety and efficacy of AS for small, low-risk PTC, even beyond microcarcinomas.

Our results should be considered in the context of the well-established body of evidence indicating that older patients are at significantly increased risk of surgical complications from thyroidectomy.^22^ Papaleontiou and colleagues^23^ reported that in US individuals older than 65 years, the odds ratios of risk of general surgical complications and thyroid-specific surgical complications were 2.61 and 3.21, respectively, compared to younger individuals. Heightened surgical risks in older individuals need to be weighed in decision-making on management of PTC.

### Strengths and Limitations

Some strengths of this study include its prospective nature, inclusion of a substantial proportion of patients with PTC larger than 1 cm in diameter, cytopathologic confirmation of the diagnosis in all cases, prospectively collected data on the decision-making process, a relatively long duration of prospective follow-up, and detailed outcome data collection. However, this study is subject to several limitations. Participants were not randomized to AS or surgery; however, randomization would have complicated the interpretation of the analyses of AS durability, since patients would not have had the opportunity to participate in making the disease management choice. Yet, the lack of randomization likely contributed to some selection bias and some differences in baseline characteristics between the AS and immediate surgical group. Second, data were collected in a single Canadian tertiary care center, which may limit the external generalizability of our findings to other health care settings. We also appreciate that there may be cultural differences among patients and their health care practitioners internationally, which could impact treatment decision-making at initial diagnosis and over time. Some secondary subgroup comparisons in this study were limited by small sample sizes and were likely underpowered to detect differences between groups (eg, the analyses comparing patients who crossed over from AS to those who had immediate surgery). However, the value of the descriptive surgical data is that they reflect the fact that our general approach to surgical treatment of patients in the study was relatively conservative and that the surgical complication rates were reasonably low. Statistical analyses were not corrected for multiple testing, which could increase the risk of type I error. Post hoc analyses, such as the analysis exploring an optimum age cutoff, should be considered hypothesis generating. Longer follow-up is desirable in evaluating outcomes of low-risk PTC, and we are continuing to follow the patients in this study as part of a larger multicenter study.^14^

## Conclusions

In conclusion, based on our findings and those published in the literature, AS is a durable, safe, long-term disease management option for patients with small, low-risk PTC. Older age may be associated with greater AS durability. We, like others,^5,10,11,17,18,19,20,21^ have observed that small, low-risk PTC AS outcomes are largely favorable, and disease progression is readily treatable. We suggest that AS may be offered as a first-line PTC management option for patients with small, low-risk tumors, and being offered this choice is especially relevant for older individuals who may want to avoid surgery. It is important to mention that in this study, a concerted effort was made to inform all patients of their options and respect their PTC management preferences from the time of initial decision-making throughout ongoing follow-up. We have previously reported that 96% of patients in our study had high decision satisfaction with their initial disease management choice.^3^ One of the important messages from this study is the value of including patients across the age spectrum in cancer disease-management decision-making and respecting their informed choices.

## Notes

Supplement 1. eTable. Comparison of the Treatments, Tumor Staging, and Treatment Outcomes of Patients Who Crossed Over From Active Surveillance (AS) to Definitive Treatment* Compared to Individuals Who Underwent Immediate Surgery

Supplement 2. Canadian Thyroid Cancer Active Surveillance Study Group (Greater Toronto Area)

Supplement 3. Data Sharing Statement
