# Supplementary material for: Long-Term Durability of Active Surveillance of Small, Low-Risk Papillary Thyroid Cancer
Source: JAMA Surg. 2025 Aug 20;160(10):1117–24. doi: 10.1001/jamasurg.2025.2957 (PMC12368792; doi:10.1001/jamasurg.2025.2957)
Supplement: Supplement 1. — eTable. Comparison of the Treatments, Tumor Staging, and Treatment Outcomes of Patients Who Crossed Over From Active Surveillance (AS) to Definitive Treatment* Compared to Individuals Who Underwent Immediate Surgery [file jamasurg-e252957-s001.pdf]

# Supplemental Online Content

Sawka AM, Ghai S, Rotstein L, et al; Canadian Thyroid Cancer Active Surveillance Study Group (Greater Toronto Area). Long-term durability of active surveillance of small, low-risk papillary thyroid cancer. *JAMA Surg*. Published online August 20, 2025. doi:10.1001/jamasurg.2025.2957

**eTable.** Comparison of the Treatments, Tumor Staging, and Treatment Outcomes of Patients Who Crossed Over From Active Surveillance (AS) to Definitive Treatment* Compared to Individuals Who Underwent Immediate Surgery

This supplemental material has been provided by the authors to give readers additional information about their work.

© 2025 Sawka AM et al. *JAMA Surgery*.

**eTable.** Comparison of the Treatments, Tumor Staging, and Treatment Outcomes of Patients Who Crossed Over From Active Surveillance (AS) to Definitive Treatment Compared to Individuals Who Underwent Immediate Surgery*

| Variable | | Active Surveillance: Cross-over to active treatment (N = 32) | Immediate Surgery (N = 45) | P-value |
|---|---|---|---|---|
| Type of first thyroid cancer surgery/treatment | Hemithyroidectomy | 26/32 (81%)* | 37/45 (82%)* | 0.86 |
| | Total thyroidectomy | 4/32 (13%) | 7/45 (16%) | |
| | Isthmectomy | 1/32 (3%) | 1/45 (2%) | |
| | Radiofrequency ablation | 1/32 (3%) | 0 (0%) | |
| Extent of nodal dissection with first thyroid cancer surgery† | None | 22/31 (71%) | 35 (78%) | 0.59 |
| | Central neck | 8/31 (26%) | 9 (20%) | |
| | Lateral and central neck | 1/31 (3%)‡ | 1 (2%)‡ | |
| Tumor (T) Staging Categoryγ | T1a | 12/29 (41%) | 18/44 (41%) | 1.00 |
| | T1b | 16/29 (55%) | 24/44 (55%) | |
| | T2 | 0 | 1/44 (2%) | |
| | T3b | 1/29 (3%) | 1/44 (2%) | |

© 2025 Sawka AM et al. *JAMA Surgery*.

| | | | | |
|---|---|---|---|---|
| **Nodal metastases (N) Categoryγ** | **N0 or Nx (clinical N0)** | 20/29 (69%) | 36/44 (82%) | 0.25 |
| | **N1a** | 8/29 (28%) | 8/44 (18%) | |
| | **N1b** | 1/29 (3%) | 0 | |
| **Total thyroidectomy completed as of last follow-up§** | | 7/32 (22%)§ | 11/45 (24%)§ | 1.00 |
| **Major complication of thyroid cancer surgery/treatment** | | 1/32 (3%)β | 3/45 (7%)β | 0.64 |
| **Radioactive iodine treatment for thyroid cancer** | | 5/32 (16%) | 4/45 (9%) | 0.48 |
| **Taking thyroid hormone treatment at last follow-upΩ** | | 20/32 (63%) | 36/45 (80%) | 0.15 |
| **Thyroid cancer recurrence or persistence after surgery or other treatment** | **No persistent or recurrent thyroid cancer** | 30/32 (94%) | 43/45 (96%) | 0.75 |
| | **Local-regional recurrence treated with additional surgery, disease-free at last follow-up** | 1/32 (3%)£ | 2/45 (4%)µ | |
| | **Persistent disease in the contralateral thyroid lobe after hemi-thyroidectomy (patient declined more surgery)** | 1/32 (3%)Ø | 0 (0%) | |

© 2025 Sawka AM et al. *JAMA Surgery*.

*Includes two patients in the AS group and 2 patients in the initial surgery group who subsequently underwent completion thyroidectomy within a year of the initial surgery as well as one patient in the AS group who is awaiting completion thyroidectomy

†One individual who underwent radiofrequency ablation in the AS cross-over group is excluded as there was no thyroid surgery.

‡Lateral neck dissection with bilateral central neck dissection was performed in the first surgery in one patient in the active surveillance group who crossed over to surgery due to lateral neck nodal disease detected during surveillance. One patient in the surgical group underwent ipsilateral central neck nodal dissection and level 3 and 4 ‘exploration’ as per the treating surgeon’s decision but there was no nodal disease ultimately resected.

γStaging after the initial complete surgical treatment reported as per the AJCC (TNM) classification system (8th edition) (reference 16). The TNM staging data includes only patients who had surgery and who had proven papillary thyroid carcinoma on histopathology. The data excludes one patient in the AS group and one in the immediate surgical group who had no evidence of thyroid cancer (diagnosis of benign thyroid nodule), one patient in the AS group who had a growing nodule meeting study progression criteria and ultimately was found to have a non-invasive follicular thyroid neoplasm with papillary-like nuclear features (NIFTP) on histopathology, as well as one patient in the AS cross-over group who had radiofrequency ablation (and thus no histopathologic specimen available for staging). None of the patients with histopathologically proven PTC were diagnosed with distant metastatic disease.

© 2025 Sawka AM et al. *JAMA Surgery*.

§Total thyroidectomy completed as of last follow-up means that the patient had one or more thyroid surgeries, including surgery for initial treatment and any treatment of persistent/recurrent disease. The number of patients who had total thyroidectomy completed as of last follow-up includes 1 patient in the active surveillance group and 2 patients in the surgical group who had recurrence of disease more than a year after their first surgery and had completion thyroidectomy performed as part of the treatment of the disease recurrence in addition to patients who had 1- or 2-stage total thyroidectomy as part of their initial surgical treatment.

βIn the patients who crossed-over from AS to surgery, one patient had paresis of the recurrent laryngeal nerve after surgery. In the patients who underwent immediate surgery, one patient had a neck hematoma requiring surgical evacuation after thyroidectomy, one patient had hypoparathyroidism and continued to require calcitriol treatment at last follow-up, and one patient had paresis of the recurrent laryngeal nerve after surgery

£Patient in the active surveillance group who initially underwent an isthmectomy for patient preference later had completion thyroidectomy and lateral neck nodal dissection for lateral neck nodal disease more than a year after the original surgery

µTwo patients in the surgical group who were treated with a hemithyroidectomy had a contralateral malignant nodule that prompted completion thyroidectomy more than a year after the initial surgery

ΩThe number of patients that were taking thyroid hormone treatment at enrollment (prior to any surgery) were 5/32 (16%) of patients in the AS cross-over group and 9/45 (20%) in

© 2025 Sawka AM et al. *JAMA Surgery*.

the surgical group and the number shown taking thyroid hormone at last follow-up includes these patients.

ØPatient with an enlarging thyroid nodule (which was not the primary papillary thyroid cancer) had a hemithyroidectomy to treat the enlarging nodule that was suspected to be malignant on biopsy and declined total or completion thyroidectomy for the primary PTC (which remains stable). The enlarging nodule that was resected was benign.

© 2025 Sawka AM et al. *JAMA Surgery*.
