# Supplementary material for: Long-Term Durability of Active Surveillance of Small, Low-Risk Papillary Thyroid Cancer
Source: JAMA Surg. 2025 Aug 20;160(10):1117–24. doi: 10.1001/jamasurg.2025.2957 (PMC12368792; doi:10.1001/jamasurg.2025.2957)
Supplement: Supplement 2. — Canadian Thyroid Cancer Active Surveillance Study Group (Greater Toronto Area) [file jamasurg-e252957-s002.pdf]

Supplemental Online Content: Nonauthor Collaborators
*First name, last name, and suffix (if applicable) are required and will appear in PubMed.

*Group Name(s): The Canadian Thyroid Cancer Active Surveillance Study Group (Greater Toronto Area)							
***First Name and Middle Initial(s)**	***Last Name**	***Suffix (eg, Jr, III)**	Academic Degrees	Institution	Location (city, state/province, country)	Role or Contribution, eg, chair, principal investigator	Group (if more than 1 Group listed in the byline) and/or Subgroup (eg, Steering Committee)
Avik	Banerjee		MD	Grand River Hospital	Kitchener, Ontario, Canada	Clinical collaborator	
Vinita	Bindlish		MD	Grand River Hospital	Kitchener, Ontario, Canada	Clinical collaborator	
Maky	Hafidh		MD	Grand River Hospital	Kitchener, Ontario, Canada	Clinical collaborator	
Jose	Prudencio		MD	Grand River Hospital	Kitchener, Ontario, Canada	Clinical collaborator	
Vinod	Bharadwaj		MD	Grand River Hospital	Kitchener, Ontario, Canada	Clinical collaborator	
Denny	Lin		MD	Guelph General Hospital	Guelph, Ontario, Canada	Clinical collaborator	
Laura	Whiteacre		MD	Humber River Hospital	Toronto, Ontario, Canada	Clinical collaborator	
Eric	Arruda		MD	Lakeridge Health	Oshawa, Ontario, Canada	Clinical collaborator	
Artur	Gevorgyan		MD	Lakeridge Health	Oshawa, Ontario, Canada	Clinical collaborator	
Marshall	Hay		MD	Independent Practice	Toronto, Ontario, Canada	Clinical collaborator	
Philip	Solomon		MD	Mackenzie Health Hospital	Richmond Hill, Ontario, Canada	Clinical collaborator	
Karen	Gomez Hernandez		MD	Mount Sinai Hospital	Toronto, Ontario, Canada	Clinical Collaborator	
Allan	Vescan		MD	Mount Sinai Hospital	Toronto, Ontario, Canada	Clinical Collaborator	
Ian	Witterick		MD	Mount Sinai Hospital	Toronto, Ontario, Canada	Clinical Collaborator	
Everton	Gooden		MD	North York General Hospital	Toronto, Ontario, Canada	Clinical Collaborator	
Manish	Shah		MD	North York General Hospital	Toronto, Ontario, Canada	Clinical Collaborator	

*First name, last name, and suffix (if applicable) are required and will appear in PubMed.

***First Name and Middle Initial(s)**	***Last Name**	***Suffix (eg, Jr, III)**	Academic Degrees	Institution	Location (city, state/province, country)	Role or Contribution, eg, chair, principal investigator	Group (if more than 1 Group listed in the byline) and/or Subgroup (eg, Steering Committee)
Michael	Chang		MD	Scarborough Health Network (Centenary Hospital)	Scarborough, Ontario, Canada	Clinical Collaborator	
Andres	Gantous		MD	St. Joseph's Health Centre	Toronto, Ontario, Canada	Clinical Collaborator	
Jennifer	Anderson		MD	St. Michael's Hospital	Toronto, Ontario, Canada	Clinical Collaborator	
Vinay	Fernandes		MD	Scarborough Health Network (General Hospital)	Scarborough, Ontario, Canada	Clinical Collaborator	
Sumeet	Anand		MD	Scarborough Health Network (Birchmount Hospital, Centenary Hospital, General Hospital)	Scarborough, Ontario, Canada	Clinical Collaborator	
Danny	Enepekides		MD	Sunnybrook Health Sciences Centre	Toronto, Ontario, Canada	Clinical Collaborator	
Antoine	Eskander		MD	Sunnybrook Health Sciences Centre	Toronto, Ontario, Canada	Clinical Collaborator	
Ilana J.	Halperin		MD	Sunnybrook Health Sciences Centre	Toronto, Ontario, Canada	Clinical Collaborator	
Kevin	Higgins		MD	Sunnybrook Health Sciences Centre	Toronto, Ontario, Canada	Clinical Collaborator	
Karim	Nazarali		MD	Trillium Health Partners	Toronto, Ontario, Canada	Clinical Collaborator	
Lorne	Segall		MD (Deceased)	Trillium Health Partners	Toronto, Ontario, Canada	Clinical Collaborator	
John	de Almeida		MD	University Health Network	Toronto, Ontario, Canada	Clinical Collaborator	
Shereen	Ezzat		MD	University Health Network	Toronto, Ontario, Canada	Clinical Collaborator	
Ralph	Gilbert		MD	University Health Network	Toronto, Ontario, Canada	Clinical Collaborator	
Patrick J.	Gullane		MD	University Health Network	Toronto, Ontario, Canada	Clinical Collaborator	
Amin	Madani		MD	University Health Network	Toronto, Ontario, Canada	Clinical Collaborator	

Supplemental Online Content: Nonauthor Collaborators
*First name, last name, and suffix (if applicable) are required and will appear in PubMed.

***First Name and Middle Initial(s)**	***Last Name**	***Suffix (eg, Jr, III)**	Academic Degrees	Institution	Location (city, state/province, country)	Role or Contribution, eg, chair, principal investigator	Group (if more than 1 Group listed in the byline) and/or Subgroup (eg, Steering Committee)
Richard W.	Tsang		MD	University Health Network		Clinical Collaborator	
Mark	Korman		MD	William Osler Health System	Brampton, Ontario, Canada	Clinical Collaborator	
Karen	Devon		MD	Women's College Hospital	Toronto, Ontario, Canada	Clinical Collaborator	
Afshan	Zahedi		MD	Women's College Hospital	Toronto, Ontario, Canada	Clinical Collaborator	
