# Supplementary material for: Long-Term Durability of Active Surveillance of Small, Low-Risk Papillary Thyroid Cancer
Source: JAMA Surg. 2025 Aug 20;160(10):1117–24. doi: 10.1001/jamasurg.2025.2957 (PMC12368792; doi:10.1001/jamasurg.2025.2957)
Supplement: Supplement 3. — Data Sharing Statement [file jamasurg-e252957-s003.pdf]

Data Sharing Statement

Sawka. Long-Term Durability of Active Surveillance of Small, Low-Risk Papillary Thyroid Cancer. *JAMA Surg*. Published August 20, 2025. doi:10.1001/jamasurg.2025.2957

Data
Data available: No

Additional Information
Explanation for why data not available: Open data sharing was not approved by our institutional ethics board for this study. The corresponding author may be contacted with any questions about the data.
